# Instrumente behördlicher Kommunikation zu Anwendungsrisiken von Arzneimitteln

**DOI:** 10.1007/s00103-022-03527-w

**Published:** 2022-04-26

**Authors:** Simone Bergner, Thomas Grüger, Martin Huber, Walburga Lütkehermölle, Norbert Paeschke, Harriet Palissa, Kerstin Stephan, Sabine Cibura, Brigitte Keller-Stanislawski

**Affiliations:** 1 Bundesinstitut für Arzneimittel und Medizinprodukte (BfArM), Kurt-Georg-Kiesinger-Allee 3, 53175 Bonn, Deutschland; 2 Presse und Öffentlichkeitsarbeit, Bundesinstitut für Arzneimittel und Medizinprodukte (BfArM), Bonn, Deutschland; 3 Paul-Ehrlich-Institut (PEI) Bundesinstitut für Impfstoffe und biomedizinische Arzneimittel, Langen, Deutschland

**Keywords:** Pharmakovigilanz, Risikokommunikation, Arzneimittel, Impfstoffe, Arzneimittelbehörden, Pharmacovigilance, Risk communication, Medicinal products, Vaccines, Drug regulatory agencies

## Abstract

Die aktive Kommunikation von Behörden, wie dem Bundesinstitut für Arzneimittel und Medizinprodukte (BfArM) und dem Paul-Ehrlich-Institut (PEI), einschließlich der Kontaktpflege zu Fachkreisen sowie Presse- und Öffentlichkeitsarbeit sind wesentliche Voraussetzung dafür, dass Informationen zu Anwendungsrisiken von Arzneimitteln sowohl betroffene Patientinnen und Patienten als auch Angehörige der Heilberufe schnell und gezielt erreichen. Die verschiedenen Instrumente der gezielten Kommunikation beschreiben mögliche Risiken und enthalten darüber hinaus auch Empfehlungen, die helfen, das Anwendungsrisiko eines Arzneimittels zu reduzieren. Die ergänzende Öffentlichkeitsarbeit zielt darauf ab, die Aufgaben und Ziele der Behörde in der Bevölkerung und in Fachkreisen bekannt zu machen, um Vertrauen in behördliches Handeln zu schaffen und auszubauen. Dafür müssen entsprechende Kommunikationsplattformen etabliert und akzeptiert sein, die sowohl von Fachkreisen als auch von der Bevölkerung genutzt werden können. Die aktuell verfügbaren Instrumente der gezielten Risikokommunikation, wie Rote-Hand-Briefe (RHB), Risikomanagementpläne und Schulungsmaterial, werden in dieser Publikation ebenso beschrieben wie die breiter angelegte Kommunikation auf den behördlichen Webseiten oder gegenüber den Medien. Schließlich wird die Risikokommunikation des PEI unter besonderer Berücksichtigung der COVID-19-Impfstoffe beleuchtet.

## Abstract (en)

Active communication of authorities, such as the Federal Institute for Drugs and Medical Devices (BfArM) and the Paul Ehrlich Institute (PEI), including maintenance of contacts with health care professionals, as well as press and public relations work, are essential prerequisites for ensuring that information on the risks of using medicinal products reaches both affected patients and healthcare professionals quickly and in a targeted manner. The various instruments of targeted communication describe possible risks and also contain recommendations that help to reduce the risk of using a medicinal product. The supplementary public relations work aims to make the tasks and objectives of the authority known to the public and to experts with the goal of creating and expanding trust in the actions of the authorities. To this end, appropriate communication platforms must be established and accepted so that they are used by both experts and the general public and the authority is perceived and appreciated as a reliable source of risk information. The currently available instruments of targeted risk communication, such as Dear Health Care Professional Communication (DHPC), risk management plans, and educational materials are described in this paper as well as broader communication on official websites or towards the media. Finally, PEI’s risk communication is highlighted with particular reference to COVID-19 vaccines.

## Einleitung

Die Aufgaben und Themen von Arzneimittelbehörden berühren die Lebenswirklichkeit der Menschen in besonderer Weise. Gesundheit und Behandlung von Krankheiten sind sehr persönliche und oft hochemotionale Themen, die in der Öffentlichkeit hohe Aufmerksamkeit erfahren. In diesem komplexen Umfeld ist eine erfolgreiche Risikokommunikation mit besonderen Herausforderungen verbunden.

Risikokommunikation ist elementarer Bestandteil der Pharmakovigilanz. Die Weltgesundheitsorganisation (WHO) hat im Jahr 2002 eine Definition des Begriffs Pharmakovigilanz publiziert: „Science and activities relating to the detection, assessment, understanding and prevention of adverse effects or any other medicine-related problem“ [1]. Zu den grundlegenden Zielen der Pharmakovigilanz zählt somit nicht nur das Erkennen und die Bewertung möglicher Risiken, sondern auch das Verstehen der Zusammenhänge, um Strategien zur Vermeidung von Anwendungsrisiken zugelassener Arzneimittel zu entwickeln. Dabei stehen nicht nur die zugelassenen Anwendungsbedingungen im Fokus, sondern auch Risiken, die sich aus der Anwendung außerhalb der Zulassungsbedingungen, im Rahmen beruflicher Exposition oder aus dem Medikationsprozess heraus ergeben können. Die Vermeidung möglicher Anwendungsrisiken setzt deren Kenntnis voraus, weshalb sowohl die Kommunikation bekannt gewordener Risiken als auch die rechtzeitige Bereitstellung von Information zu deren möglicher Reduzierung zu den Kernaufgaben der Pharmakovigilanz gehören. Die Risikokommunikation hat dazu einen breiten Adressatenkreis im Blick und dient hier dem vorbeugenden Gesundheitsschutz von Patientinnen und Patienten.

Die Kommunikationskanäle umfassen sowohl allgemein verfügbare Informationen auf der behördlichen Webseite als auch Materialien, die den betroffenen Patientinnen und Patienten ggf. gezielt ausgehändigt werden. Diese arzneimittelbezogene Kommunikation wird ergänzt durch die Presse- und Öffentlichkeitsarbeit, die übergeordnete Aspekte der aktiven behördlichen Kommunikation adressiert, aber auch Fragestellungen aus dem öffentlichen bzw. medialen Raum aufgreift.

Risikokommunikation findet – im Gegensatz zur Krisenkommunikation – im Vorfeld einer Krise statt. Ihre Aufgabe ist es, die Risikowahrnehmung und das Verhalten von Menschen zu beeinflussen, um gesellschaftlichen Schaden zu begrenzen oder zu reduzieren. Menschen sollen in die Lage versetzt werden, auf Grundlage entsprechender Informationen fundierte Entscheidungen zu treffen.

Damit dies im Fall einer Krise auch gelingt, muss sich der Absender solcher Informationen in der öffentlichen Wahrnehmung bereits vor der Krise als vertrauenswürdig etablieren. Nur dann werden seine Botschaften im Krisenfall ebenfalls als vertrauenswürdig wahrgenommen. Für das Bundesinstitut für Arzneimittel und Medizinprodukte (BfArM) und das Paul-Ehrlich-Institut (PEI) bedeutet dies beispielsweise, Patientinnen und Patienten nicht nur über den Nutzen, sondern immer auch über die Risiken von Arzneimitteln transparent zu informieren. Ebenso müssen Probleme umgehend kommuniziert werden – auch wenn noch nicht alle Details des Sachverhaltes zu diesem Zeitpunkt bekannt sind. Indem die Behörde hier frühestmöglich den Dialog aufnimmt und die Adressaten in jeden weiteren Schritt der Entwicklung einbindet, schafft sie Vertrauen in ihre Kommunikation. Dieses Vorgehen ist sowohl für die Risiko- als auch für die Krisenkommunikation von besonderer Bedeutung.

## Gesetzliche Grundlagen

Die Europäische Union (EU) hat verschiedene Instrumente entwickelt, die eine risikoadaptierte Kommunikation erlauben. Die verschiedenen Instrumente sind gesetzlich verankert. Das BfArM und das PEI sind in das europäische Netzwerk der zuständigen nationalen Behörden für die Arzneimittelzulassung und Pharmakovigilanz eingebunden. Durch die zunehmende europäische Harmonisierung von Zulassungsentscheidungen und von Maßnahmen zur Minimierung von Anwendungsrisiken bei Arzneimitteln wird sichergestellt, dass Risikoinformationen innerhalb der EU weitgehend inhaltsgleich zur Verfügung gestellt werden und sich i. d. R. nur punktuell im Hinblick auf zu beachtende nationale Besonderheiten des jeweiligen Gesundheitssystems unterscheiden.

Grundlage für die Regelungen nach dem Arzneimittelgesetz (AMG; [2]) ist die europäische Gesetzgebung basierend auf der Verordnung [EG] Nr. 726/2004 in Verbindung mit der Richtlinie 2001/83/EG [3] sowie auf den europäischen Leitlinien des Good Vigilance Practice Guide ([4]; GVP, insbesondere GVP-Modul V [5], XV [6] und XVI [7]) sowie der Summary of Product Characteristics (SmPC) Guideline [8]. Im AMG sind die europäischen Regelungen in den §§ 11 und 11a (Packungsbeilage und Fachinformation) sowie im § 34 (Information der Öffentlichkeit) umgesetzt worden. Dazu gehören nach § 34 Abs. 1a AMG u. a. die Einrichtung eines Internetportals, die Publikation der Zusammenfassungen von Risikomanagementplänen sowie allgemein die Veröffentlichung von Bedenken aus dem Bereich der Pharmakovigilanz. Zu Letzteren gehört die i. d. R. EU-weit abgestimmte „Direct Healthcare Professional Communication“ (DHPC, umgangssprachlich „Dear Doctor Letter“). Die DHPC ist in Deutschland in Form der sog. Rote-Hand-Briefe seit vielen Jahren etabliert. § 34 Abs. 1f eröffnet ergänzend die Möglichkeit, auch behördlich angeordnetes und genehmigtes Schulungsmaterial über ein Portal zur Verfügung zu stellen, was in Deutschland über die Webseiten von BfArM^1^ und PEI^2^ umgesetzt ist.

## Möglichkeiten behördlicher Kommunikation zu Risiken bei der Anwendung von Arzneimitteln

### Fachinformation (FI) und Gebrauchsinformation (GI)

Die Einnahme von Arzneimitteln birgt, trotz allem Nutzen, unweigerlich gewisse Risiken in sich. Vielfach ist dieses Risiko vernachlässigbar, jedoch ist es sowohl für die Patientinnen und Patienten als auch für die Angehörigen der Heilberufe vor dem Hintergrund der zugelassenen Anwendungsgebiete von großer Bedeutung, über sämtliche Eigenschaften, identifizierte Risiken, Warn- und Anwendungshinweise sowie Wechselwirkungen vor Verschreibung und Anwendung verständlich informiert zu werden. Gebrauchsinformationen (GI) für Patientinnen und Patienten (Packungsbeilagen) und Fachinformationen (FI) für Fachkreise geben Auskunft, wie ein Arzneimittel bestimmungsgemäß angewendet werden soll. Dabei können Patientinnen, Patienten und Angehörige des Gesundheitswesens auf standardisierte Informationsformate zu Arzneimitteln (sog. QRD-Templates [9]) zurückgreifen. Pharmazeutische Unternehmen sind nach dem AMG verpflichtet, FI und GI routinemäßig zu erstellen und regelmäßig an den jeweils aktuellen wissenschaftlichen Stand anzupassen. Diese Aufgabe erfordert, dass auch die Gesundheitsbehörden wie das BfArM und das PEI sämtliche zugelassene Arzneimittel regelmäßig überwachen und bei Bedarf Aktualisierungen anordnen. Die Produktinformationstexte sind dabei national oder europäisch durch das Netzwerk der Zulassungsbehörden der Mitgliedsländer und der Europäischen Arzneimittelagentur (EMA) wissenschaftlich geprüft und genehmigt.

Diese Informationstexte sind gleichzeitig wichtige Instrumente zur Kommunikation von Arzneimittelrisiken sowie zur Beschreibung risikominimierender Maßnahmen. Sie erlauben den Anwenderinnen und Anwendern daher die Abwägung von Nutzen und potenziell schädlichen Wirkungen entsprechend dem jeweiligen aktuellen wissenschaftlichen Kenntnisstand und erlauben, Anwendungsnutzen und potenzielle Risiken vor Verordnung und Anwendung hinreichend zu berücksichtigen. Dabei ist die Kommunikation der Inhalte von FI und GI in einer geeigneten Sprache für die jeweilige Zielgruppe von besonderer Bedeutung. Dabei ergeben sich mitunter Zielkonflikte bei der Gestaltung der Produktinformationstexte in ihrer bisherigen Papierform, da diese neben den Anforderungen an die Aktualität auch denen der europaweiten Standardisierung und Textaufbereitung genügen müssen.

Durch die zunehmende Nutzung digitaler Endgeräte und damit einhergehend digitaler Angebote werden auch Produktinformationstexte immer häufiger zusätzlich auf digitalem Wege online bzw. per App (z. B. europaweites Projekt GI 4.0; [10, 11]) bereitgestellt. Die wesentlichen Vorteile gegenüber der Papierversion sind die Aktualisierung der Texte, die elektronischen Möglichkeiten der Strukturierung und Lesbarkeit sowie des gezielten Zugriffs auf einzelne Inhalte. Integrierte Barcodes ermöglichen die einfache Verlinkung auf ergänzende Informationen.

### Rote-Hand-Briefe (RHB)

Die Erstellung und Versendung von sog. Rote-Hand-Briefen auf nationaler Ebene oder von Direct Healthcare Professional Communications auf europäischer Ebene folgt den EU-Vorgaben der Richtlinien für „Gute-Pharmakovigilanzpraxis“ GVP-Modul XV – „Safety Communication“ [6]. Als Mittel der Risikokommunikation informieren sie Ärztinnen, Ärzte, Apothekerinnen und Apotheker, aber auch Pflegepersonal oder Therapeutinnen sowie Therapeuten schnell und zielgerichtet über neue, bisher nicht bekannte Arzneimittelanwendungsrisiken oder therapierelevante Änderungen des Risikoprofils. Dabei kann es sich z. B. um eine neue Kontraindikation, neue Warnhinweise oder einen Rückruf der Arzneimittelzulassung aufgrund eines Qualitätsmangels handeln. Der pharmazeutische Unternehmer ist gemäß § 11a Abs. 2 AMG verpflichtet, diese Informationen zur Verfügung zu stellen [2]. Häufig stammen die Informationen aus Ergebnissen von Verfahren zur Risikobewertung (sog. Referrals), der Beurteilung von periodischen Sicherheitsberichten oder der Bewertung von Signalen zu neuen oder veränderten Risiken.

**Figure 1:**
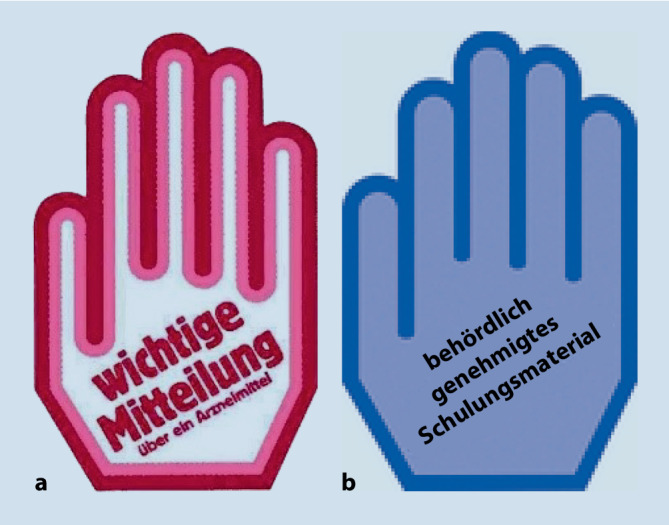

Rote-Hand-Briefe unterscheiden sich von sog. Informationsbriefen. Letztere informieren die Fachkreise über relevante Änderungen zum Risikoprofil, die aber nicht unmittelbar therapierelevant sind. Sie tragen nicht das für RHB übliche Rote-Hand-Symbol (Abb. 1).

Nach dem AMG müssen RHB anders als „Informationsbriefe“ von pharmazeutischen Unternehmen mit der jeweils zuständigen Landesbehörde oder Bundesoberbehörde inhaltlich und bezüglich des Adressatenkreises abgestimmt werden. Diese Vorgabe stellt die Objektivität der Inhalte sicher und verhindert irreführende oder werbende Formulierungen. Der Versand selbst erfolgt eigenverantwortlich durch das pharmazeutische Unternehmen, bei mehreren beteiligten Unternehmen auch koordiniert durch die Verbände der pharmazeutischen Industrie. Perspektivisch sollen RHB verpflichtend in elektronischer Form innerhalb von Praxissoftware zur Verfügung gestellt werden. Die gesetzlichen Voraussetzungen im Fünften Buch Sozialgesetzbuch (SGB V; [12]) liegen mittlerweile vor (vgl. Abschnitt Schulungsmaterial). Die digitale Zurverfügungstellung der RHB wird auch mit der nationalen Agentur für Digitale Medizin (gematik) im Rahmen der Telematikinfrastruktur diskutiert.

### Risikomanagementsystem und Risikomanagementplan

Zum Zeitpunkt der Zulassung eines Arzneimittels sind die Daten aus der klinischen Entwicklung in der Regel begrenzt. So sind bestimmte Anwendergruppen u. a. aus ethischen Gründen oft nicht hinreichend in den Zulassungsstudien vertreten. Aus methodischen Gründen ist es zudem nahezu unmöglich, alle Anwendungsrisiken zum Zeitpunkt der Zulassung zu ermitteln bzw. zu beschreiben. Nach dem AMG und den eingangs genannten und seit Juli 2012 geltenden europäischen Gesetzesgrundlagen sind die Zulassungsinhaber verpflichtet, für jedes neu zugelassene Arzneimittel ein sogenanntes Risikomanagementsystem zu betreiben [13, 14]. Dies gilt unter bestimmten Voraussetzungen auch für bereits vor diesen Stichtagen zugelassene Arzneimittel. Das Risikomanagementsystem (RMS) beschreibt Tätigkeiten und Maßnahmen zur zeitnahen Aufklärung von Wissenslücken und bedeutsamen Anwendungsrisiken, z. B. bezüglich der Häufigkeit, Schwere und Risikofaktoren, aber auch, wie bedeutsame Risiken vermieden oder zumindest minimiert werden sollen. „Bedeutsame Risiken und Wissenslücken“ sind so definiert, dass sie einen maßgeblichen Einfluss auf das Nutzen-Risiko-Verhältnis haben oder sich nach weiterer Abklärung ein solcher Einfluss ergeben könnte.

Eine weitergehende Charakterisierung dieser Risiken bzw. Wissenslücken erfordert oft Maßnahmen, die über die routinemäßige Pharmakovigilanz hinausgehen. Dabei stellen Unbedenklichkeitsprüfungen nach der Zulassung (Post-Authorisation Safety Study: PASS) oft ein effizientes Instrument dar. PASS dienen auch der Bewertung, ob zusätzlich getroffene Maßnahmen zur Risikominimierung wie Schulungsmaterialien oder Rote-Hand-Briefe hinreichend wirksam gewesen sind.

**Figure 2:**
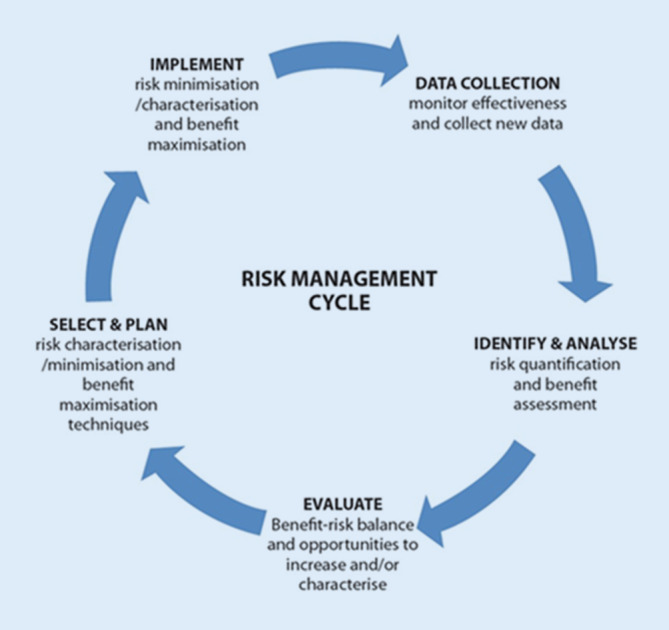

Die detaillierte Beschreibung dieses RMS ist der Risikomanagementplan (RMP; [15, 16]). Der RMP ist somit ein wichtiges Instrument und integraler Bestandteil jeder neuen Arzneimittelzulassung in Deutschland und der EU. Er wird während des gesamten Lebenszyklus des Arzneimittels entsprechend den Erkenntnissen aus den durchgeführten Untersuchungen oder auch neu bekannt gewordenen Anwendungsrisiken aktualisiert (Abb. 2).

Einzelheiten zu Struktur und Inhalt eines RMP sind im Modul V des EU-Leitfadens zur Guten Pharmakovigilanzpraxis (GVP-Modul V) festgelegt und wurden auch jüngst im Rahmen einer Publikation im Bulletin für Arzneimittelsicherheit näher erläutert [18].

Die Zusammenfassungen der RMP werden auf der Webseite der EMA (www.ema.europa.eu) für zentral zugelassene Arzneimittel bzw. des BfArM und des PEI für nicht zentral zugelassene Arzneimittel publiziert und sind somit Bestandteil der Risikokommunikation der EMA bzw. des BfArM und des PEI.

### Pharmakovigilanzforschung

Ergänzend zu den gegenüber pharmazeutischen Unternehmen angeordneten Studien werden vor allem bei wirkstoff- bzw. arzneimittelgruppenübergreifenden Fragestellungen Studien von den Überwachungsbehörden initiiert. EU-weit betrachtet werden Forschungsprojekte zum Risikobewusstsein und zur Adhärenz gefördert, um u. a. Unterschiede in der Erreichung der Zielgruppen in Europa zu untersuchen [19].

Externe Pharmakovigilanzforschungsprojekte, die vom BfArM gefördert werden, fokussieren sich dabei auf die Situation in Deutschland. Die Ergebnisse ermöglichen eine Beurteilung, ob eine Adaptierung der Risikokommunikation in Deutschland notwendig erscheint. Eine Auswahl dieser Projekte wird in Tab. 1 aufgeführt.

**Table 1:** 

Projekttitel	Projektnehmer	Weiterführende Informationen
Entwicklung der Verordnungsweise von kombinierten hormonalen Kontrazeptiva vor und nach dem EMA Risikobewertungsverfahren 2013/2014 auf der Basis von Krankenkassendaten	PMV forschungsgruppe an der Klinik und Poliklinik für Kinder- und Jugendpsychiatrie, Medizinische Fakultät, Universität zu Köln	[20, 21]
Analyse der Verordnungsweise von Valproat und Valproat-verwandten Substanzen sowie anderen oralen Antiepileptika im zeitlichen Trend mit besonderem Fokus auf Frauen im gebärfähigen Alter auf der Basis von Krankenkassendaten	PMV forschungsgruppe an der Klinik und Poliklinik für Kinder- und Jugendpsychiatrie, Medizinische Fakultät, Universität zu Köln	[22]
Überprüfung der Effektivität von Risikominimierungsmaßnahmen bei teratogenen (Fehlbildungen hervorrufenden) Substanzen in der Schwangerschaft	Pharmakovigilanz- und Beratungszentrum für Embryonaltoxikologie, Charité – Universitätsmedizin Berlin	[23]
Verordnung von teratogenen Substanzen bei Frauen im gebärfähigen Alter auf der Basis von Krankenkassendaten	Leibniz-Institut für Präventionsforschung und Epidemiologie – BIPS GmbH, Bremen	[24]

*BfArM* Bundesinstitut für Arzneimittel und Medizinprodukte, *EMA* Europäische Arzneimittelagentur

### Schulungsmaterial – zusätzliches Informationsformat zur Risikominimierung neben Fach- und Gebrauchsinformationen

Fach- und Gebrauchsinformationen sind allein nicht immer ausreichend, um Risiken bei der Anwendung von Arzneimitteln zu minimieren. In bestimmten Fällen wird daher die Erstellung sogenannten Schulungsmaterials (Educational Material) durch den Zulassungsinhaber als zusätzliche, im RMP (s. oben) dokumentierte, risikominimierende Maßnahme angeordnet und in Abstimmung mit der zuständigen Bundesoberbehörde erstellt. Die Bereitstellung bzw. Verwendung des genehmigten Schulungsmaterials ist Voraussetzung für ein rechtmäßiges Inverkehrbringen des Arzneimittels.

Der strukturelle Aufbau und die Zweckbestimmung des einzureichenden Schulungsmaterials richtet sich nach den Vorgaben des GVP-Moduls XVI („risk minimisation measures: selection of tools and effectiveness indicators“). Die Kernelemente, auf deren Basis das Schulungsmaterial zu erstellen ist, werden auf Grundlage der im RMP (GVP Modul V) beschriebenen bedeutsamen Risiken (Safety Concerns) dargelegt. Schulungsmaterial kann unabhängig vom RMP auch eigenverantwortlich durch den Zulassungsinhaber erstellt werden. Allerdings trägt nur angeordnetes und von den Behörden geprüftes Schulungsmaterial regelmäßig das sog. Blaue Hand-Symbol ([25]; Abb. 1), welches 2016 eingeführt worden ist.

**Figure 3:**
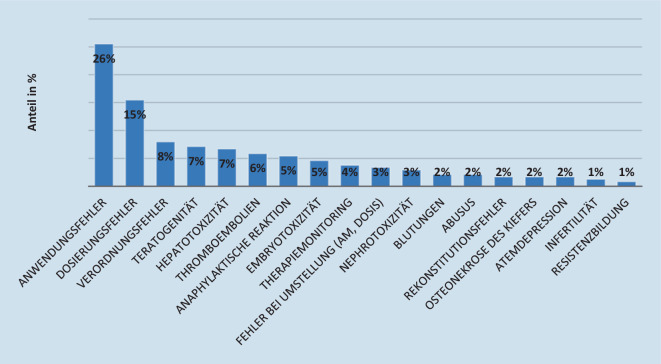

Schulungsmaterial wird zielgruppenorientiert für Patientinnen, Patienten und Angehörige der Heilberufe erstellt und fokussiert auf die Minimierung von besonders bedeutsamen Risiken, deren Vermeidung in der medizinisch-pharmazeutischen Praxis streng zu beachten ist. Eine im BfArM im Jahr 2018 durchgeführte Analyse [26] zeigte, dass Schulungsmaterialien größtenteils folgende Themen adressieren: Medikationsfehler ([27]; nicht angemessen beachtete Dosierungs‑, Anwendungs‑, Verordnungshinweise, Vorgaben zum Therapiemonitoring, Umstellungs‑, Rekonstitutionshinweise; [28, 29]), durchzuführende Laborwertkontrollen, Wechselwirkungen oder die Gefahren von Überdosierungen (Abb. 3). Eine besondere Bedeutung kommt sogenannten Schwangerschaftsverhütungsprogrammen zu, bei denen Patientinnen und ggf. deren Partner über das teratogene (Fehlbildungen hervorrufende) Potenzial des jeweiligen Wirkstoffs ausdrücklich informiert werden und die Notwendigkeit der Schwangerschaftsverhütung zu betonen ist. Einen weiteren Schwerpunkt bilden erklärungsbedürftige Applikationsarten, die bei Nichtbeachtung Medikationsfehler nach sich ziehen können.

Schulungsmaterial muss als Instrument der Risikokommunikation im medizinischen und pharmazeutischen Praxisablauf umfassend zugänglich sein. Sämtliche Schulungsmaterialien werden auf den Webseiten der verantwortlichen pharmazeutischen Unternehmer sowie auf den Webseiten von BfArM und PEI gem. § 34 Abs. 1f AMG veröffentlicht [30, 31]. Trotz Publikation auf den behördlichen Webseiten liegt die arzneimittelrechtliche Verpflichtung zur Verteilung der Schulungsinformationen jedoch beim pharmazeutischen Unternehmen, das dem BfArM oder dem PEI einen zu genehmigenden „Kommunikationsplan“ vorzulegen hat, in dem die Verteilungsmodalitäten adressatengerecht festgelegt werden.

Schulungsmaterialien im Printformat müssen derzeit bei Neueinführung eines schulungsmaterialpflichtigen Arzneimittels sowie später auf Anforderung postalisch verbreitet werden, auch dann, wenn sie elektronisch zur Verfügung stehen. Weitere Kommunikationsformate sind: Schulungsvideos und -audios, Präsenzschulungen durch den pharmazeutischen Außendienst.

Aufgrund der vom BfArM und weiteren Beteiligten kürzlich angestoßenen Änderung des SGB V, § 73 Abs. 9 und § 34 Abs. 1f AMG [32] wird die Verfügbarkeit von Schulungsmaterial in Praxissoftwaresystemen voraussichtlich ab dem 01.07.2023 verpflichtend vorgeschrieben, was als wichtiger Schritt im Sinne einer weiteren Verbesserung der Sicherheit von Patientinnen und Patienten anzusehen ist.

### Nationale Risikokommunikation und Abstimmung mit der EU

Die Risikokommunikation von BfArM und PEI findet nicht allein und isoliert auf nationaler Ebene statt, sondern ist eingebunden in das Netzwerk der europäischen Arzneimittelbehörden. Für Fragen zur Sicherheit von Humanarzneimitteln wurde 2012 bei der EMA der Ausschuss für Risikobewertung im Bereich der Pharmakovigilanz (Pharmacovigilance Risk Assessment Committee – PRAC) etabliert. Gemäß Artikel 61a der Verordnung (EG) Nr. 726/2004 (in der geänderten Fassung) gehört die Kommunikation der Risiken von Nebenwirkungen zu seinen expliziten Aufgaben. Die Koordination hierfür obliegt der EMA und erfolgt in erster Linie über deren Webseite.

Jeweils am ersten Sitzungstag wird die Tagesordnung auf der Webseite der EMA veröffentlicht, die u. a. über zur Diskussion stehende mögliche neue Arzneimittelrisiken informiert. Im Regelfall am Freitag nach der Ausschusssitzung wird in Form der sog. Meeting Highlights über diskutierte Themen von besonderem öffentlichen Interesse berichtet, z. B. zum Abschluss eines Referrals, zu Empfehlungen, die aus Signalverfahren resultieren, oder zu Empfehlungen zum Versand von DHPC bzw. RHB (s. oben). Weitere Details und Hintergründe werden in Form von Sitzungsberichten (Meeting Minutes) nach deren Verabschiedung durch das PRAC veröffentlicht. Diese regelmäßigen Veröffentlichungen werden anlassbezogen ergänzt durch Ad-hoc-Risikokommunikation zu tagesaktuellen Themen, wie z. B. jüngst zu den Impfstoffen gegen COVID-19.

Inhalt und Zeitpunkt einer Risikokommunikation durch die EMA werden vorab mit den beteiligten nationalen Behörden abgestimmt mit dem Ziel, eine EU-weit gleichzeitige Kommunikation und konsistente Informationen zu allen auf der europäischen Ebene diskutierten Themen sicherzustellen.

Die europäische Kommunikation wird entsprechend von BfArM und PEI aufgegriffen und hinsichtlich der nationalen Bedürfnisse und der Situation in Deutschland adaptiert.

### Webseite als Informationsquelle

Die Webseite des BfArM (www.bfarm.de) stellt nach wie vor die wichtigste Informationsquelle über die Behörde und ihre Aufgaben dar. Auf mehr als 35.000 Seiten werden hier Informationen und verschiedene Services angeboten. Die Webseite bietet dem Bundesinstitut die Möglichkeit, Inhalte bereitzustellen, die auf die Bedürfnisse seiner Zielgruppen abgestimmt sind. Deren benötigte Informationen sind in den meisten Fällen fachlich-regulatorischer Natur.

Neben Fachgruppen nutzen aber auch immer mehr Bürgerinnen und Bürger die Seite als Informationsquelle. Ihr Anteil an den Nutzerinnen und Nutzern der BfArM-Webseite hat sich von 7 % in 2011 auf 17 % in 2019 erhöht. Diese Entwicklung zeigt auch, dass die Themen des BfArM in den vergangenen Jahren in besonderem Maße in der Öffentlichkeit präsent waren. Als im Zusammenhang mit der Maskenpflicht während der Coronapandemie das BfArM verstärkt Fragen rund um die richtige Nutzung der Masken erreichten, wurde auch hierzu eine eigene Webseite mit entsprechenden Hinweisen erstellt. Sie wurde in der Spitzenzeit (Januar 2021) rund 1,6 Mio. Mal aufgerufen, die Zugriffszahlen lagen in den übrigen Monaten jeweils bei rund 50.000.

Grundsätzlich wird das Internet verstärkt nach Informationen zu Krankheitsbildern oder Gesundheitsthemen durchsucht und die Suchergebnisse beeinflussen wiederum ganz konkret das Handeln der Bevölkerung [33]. Das BfArM begegnet diesem Anspruch unter anderem mit einem eigenen Bereich für Bürgerinnen und Bürger auf der Seite. Dort werden grundsätzliche Themen und Aufgaben laienverständlich erklärt [34].

### Risikokommunikation in Medien

Die Nutzung von Kanälen in sozialen Medien (Social Media) im Zusammenhang mit der Risikokommunikation folgt den grundsätzlich gleichen Regeln wie die Risikokommunikation allgemein: Auch hier gilt es, Vertrauen in den Absender der Informationen zu schaffen und die Plattform als Quelle für verlässliche Informationen zu etablieren. Social-Media-Plattformen wie Twitter bringen hier jedoch zusätzlich eine eigene Dynamik mit. Sie bieten den Nutzerinnen und Nutzern die Möglichkeit, direkt in Kontakt mit dem Absender der Informationen zu treten. So können öffentlich sichtbar Fragen gestellt, Kritik geäußert, aber auch Falschaussagen gepostet werden. Ein entsprechendes Communitymanagement auf den Kanälen ist daher besonders wichtig. Nicht zuletzt können Social-Media-Kanäle dazu genutzt werden, Fragen und Themen zu antizipieren und daraus eine eigene Kommunikationsstrategie zu entwickeln.

Von der Behörde wird erwartet, dass sie möglichst schnell auf entsprechende Kommentare reagiert. Insbesondere in kritischen Situationen gilt: Man kann nicht nicht kommunizieren. Langes Schweigen kann als Zeichen mangelnder Kooperation verstanden werden, Misstrauen erzeugen und für weitere negative Posts sorgen. Bleiben gar Falschaussagen unkommentiert, besteht schnell die Gefahr, die Deutungshoheit über die eigenen Botschaften zu verlieren.

Das große Interesse an den Informationen zur Nutzung von Masken zum Schutz gegen das Coronavirus wurde bereits angesprochen. Bei der Eindämmung des Infektionsgeschehens kam es besonders darauf an, die Bevölkerung vom Nutzen präventiver Maßnahmen wie dem Tragen einer Alltagsmaske zu überzeugen. Das BfArM nutzte dazu neben ausführlichen Informationen auf seiner Webseite den Twitter-Kanal, wo Grafiken und kurze Erklärungen gepostet wurden. Die Tweets zur Verwendung von Mund-Nasen-Bedeckungen, medizinischen Gesichtsmasken sowie partikelfiltrierenden Halbmasken (FFP-Masken) gehörten dabei zu den am meisten geteilten und kommentierten des Jahres 2020. Über Twitter wurden zahlreiche Fragen zur Verwendung der Masken, zu gesetzlichen Bestimmungen, medizinischem Nutzen, Risiken oder auch zum Inverkehrbringen der Masken gestellt und beantwortet. Nicht in allen Fällen war das BfArM hier der richtige Ansprechpartner. Dies wurde auch so kommuniziert und z. B. auf die Zuständigkeiten anderer Behörden verwiesen. Ziel war es, hier in einen offenen und transparenten Austausch zu treten.

Nicht zuletzt für mediales Interesse sorgte die in sozialen Medien verbreitete Aussage, das BfArM habe erklärt, dass von den Masken keine nachgewiesene Schutzwirkung ausgehe („Bundesinstitut bestätigt: Mund-Nasen-Bedeckung hat keine Schutzwirkung!“). Als Reaktion darauf postete das BfArM neben der Beantwortung zahlreicher Fragen auch eine Richtigstellung, die von zahlreichen Nutzerinnen und Nutzern geteilt und „gelikt“ wurde.

### Bulletin zur Arzneimittelsicherheit

Das Bulletin zur Arzneimittelsicherheit dient der Kommunikation der Pharmakovigilanzaktivitäten des BfArM und des PEI. Es beinhaltet wissenschaftliche Beiträge zu Arzneimittelrisiken und Methoden der Pharmakovigilanz sowie zum regulatorischen Hintergrund der Pharmakovigilanzaktivitäten beider Behörden. Darüber hinaus wird über Risikoinformationen der nationalen und europäischen Behörden berichtet. Das Erscheinen der Publikation geht auf eine Initiative im Rahmen des Aktionsplans zur Verbesserung der Arzneimitteltherapiesicherheit in Deutschland des Bundesministeriums für Gesundheit zurück [35]. Es richtet sich in Sprache und Darstellung an Patientinnen und Patienten, die interessierte Öffentlichkeit und die Angehörigen der Heilberufe. Das Bulletin erscheint vierteljährlich in gedruckter und elektronischer Form [36].

## Risikokommunikation des PEI unter besonderer Berücksichtigung der COVID-19-Impfstoffe

Ende Dezember 2019 berichtete die Volksrepublik China der Weltgesundheitsorganisation (WHO) über ungewöhnliche Fälle einer Pneumonie in Wuhan [37, 38], wobei sich schnell herausstellte, dass es sich um ein neues Coronavirus (SARS-CoV-2) handelt. Am 21.12.2020 wurde der erste mRNA-Impfstoff (Comirnaty, BioNTech Manufacturing GmbH) nach positiver Bewertung durch den Ausschuss für Humanarzneimittel (CHMP) der EMA durch die Europäische Kommission zugelassen, gefolgt von 4 weiteren hochwirksamen COVID-19-Impfstoffen (Spikevax®, Biotech Spain, S. L.; Vaxzevria®, AstraZeneca, COVID-19 Vaccine Janssen®, Janssen-Cilag International NV) und seit 20.12.2021 Nuvaxovid (Novavax CZ, a. s.), der bislang aber in der EU noch nicht vermarktet wurde (Stand 31.12.2021). Zur Zulassung wurden klinische Prüfungen mit ca. 25.000 bis 44.000 Teilnehmerinnen und Teilnehmern eingereicht.

**Figure 4:**
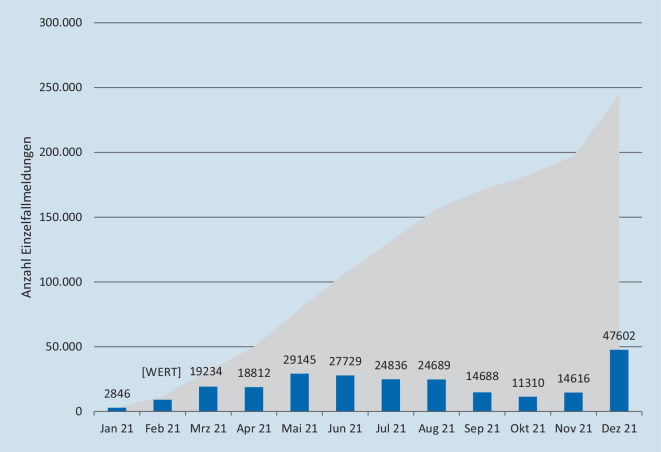

Unter Berücksichtigung der raschen Impfung großer Bevölkerungsgruppen besteht für die Pharmakovigilanz die besondere Herausforderung, dass innerhalb kürzester Zeit eine große Menge sicherheitsrelevanter Informationen bearbeitet, prozessiert und bewertet werden muss. Bis zum 30.12.2021 wurden laut Angaben des Robert Koch-Instituts (RKI) 148.760.720 Impfungen durchgeführt, davon 110.533.639 Impfungen mit Comirnaty, 21.912.123 Impfungen mit Spikevax, 12.738.494 Impfungen mit Vaxzevria und 3.576.464 Impfungen mit COVID-19 Vaccine Janssen. Insgesamt wurden 244.576 Verdachtsfälle einer Nebenwirkung nach Comirnaty, Spikevax, Vaxzevria und COVID-19 Vaccine Janssen gemeldet (Abb. 4). Die Melderate betrug für diese Impfstoffe zusammen 1,64 Meldungen pro 1000 Impfdosen und für schwerwiegende Reaktionen 0,20 Meldungen pro 1000 Impfdosen. Im Vergleich wurden im gesamten Jahr 2018 zu allen zugelassenen Impfstoffen 3570 Nebenwirkungsverdachtsfälle berichtet. Bedingt durch die erhöhte Aufmerksamkeit in der Bevölkerung in Hinblick auf die zugelassenen COVID-19-Impfstoffe ist die Melderate von Verdachtsfällen von Nebenwirkungen deutlich höher und nicht vergleichbar mit anderen Impfstoffen.

Neben den Verdachtsmeldungen zu Nebenwirkungen, einer zentralen Säule für die Beurteilung der Sicherheit von Arzneimitteln, wertet das PEI fortlaufend Publikationen, Studienergebnisse sowie präklinische und experimentelle Daten aus, um eine kontinuierliche Beurteilung von Nutzen und Risiko zu gewährleisten.

Das Thema Impfung ist nicht immer leicht zu vermitteln, da es sowohl um das individuelle Wohl jedes Einzelnen als auch um das Allgemeinwohl geht. Eine transparente, zeitnahe und gut verständliche evidenzbasierte Information der Daten zur Wirksamkeit und Sicherheit der COVID-19-Impfstoffe ist dabei essenziell und kann dazu beitragen, das Vertrauen der Bevölkerung in die Gesundheitsbehörden und die Impfkampagne zu stärken [39]. Eine offene Kommunikation über Wirksamkeit und Sicherheit eines Impfstoffs sollte auch dazu dienen, Mitbürgerinnen und Mitbürger in die Lage zu versetzen, eine fundierte Impfentscheidung zu treffen. Dazu gehört auch, Informationen zu präsentieren, selbst wenn die Fakten noch nicht vollständig geklärt sind. Gleichzeitig sollten falsche Informationen und unbegründete Ängste auf der Basis der vorhandenen Evidenz korrigiert werden.

Dabei nutzt das PEI verschiedene Formate, von denen einzelne ausgesuchte Beispiele kurz beschrieben werden:

### Sicherheitsberichte.

Seit Beginn der Impfkampagne am 27.12.2020 veröffentlicht das Paul-Ehrlich-Institut in regelmäßigen Abständen eine Zusammenfassung der wissenschaftlichen Erkenntnisse zur Pharmakovigilanz der COVID-19-Impfstoffe mit Schwerpunkt auf Meldungen von Nebenwirkungen und Impfkomplikationen, die das PEI sowohl von Impflingen oder deren Angehörigen als auch Ärztinnen, Ärzten, Apothekerinnen und Apothekern, Gesundheitsämtern sowie den Arzneimittelkommissionen der deutschen Ärzteschaft und der Apotheker erhält. Inzwischen (Stand 31.01.2021) hat das PEI auf seiner Homepage (www.pei.de) 16 Sicherheitsberichte veröffentlicht, wobei zu beachten ist, dass nicht automatisch von einem zeitlichen auch auf einen ursächlichen Zusammenhang zwischen Impfung und unerwünschter Reaktion geschlossen werden kann.

### Veröffentlichung von Rote-Hand-Briefen auf der PEI-Homepage.

Nach § 11a Abs. 2 AMG ist „der pharmazeutische Unternehmer verpflichtet, Änderungen der Fachinformation, die für die Therapie relevant sind, den Fachkreisen in geeigneter Form zugänglich zu machen. Die zuständige Bundesoberbehörde kann, soweit erforderlich, durch Auflage bestimmen, in welcher Form die Änderungen allen oder bestimmten Fachkreisen zugänglich zu machen sind.“ Dies geschieht zumeist in Form eines Rote-Hand-Briefes. Eine Übersicht über Rote-Hand-Briefe nach COVID-19-Impfstoffen (Stand 31.12.2021) gibt Tab. 2.

**Table 2:** 

Datum	Impfstoff	Nebenwirkung
13.10.2021	COVID-19 Vaccine Janssen	Immunthrombozytopenie und venöse Thrombosen
13.10.2021	Vaxzevria	Immunthrombozytopenie mit und ohne Blutungen
19.07.2021	COVID-19 Vaccine Janssen	Kontraindikation Capillary Leak Syndrome (CLS) und neue Information zu Thrombose-mit-Thrombozytopenie-Syndrom (TTS)
19.07.2021	Comirnaty + Spikevax	Myo‑/Perikarditis
23.06.2021	Vaxzevria	Kontraindikation CLS
02.06.2021	Vaxzevria	Thrombose-mit-Thrombozytopenie-Syndrom (TTS) aktualisiert
26.04.2021	COVID-19 Vaccine Janssen	Thrombose-mit-Thrombozytopenie-Syndrom (TTS)
23.04.2021	Vaxzevria	Thrombose-mit-Thrombozytopenie-Syndrom (TTS)
13.04.2021	Vaxzevria	Thrombose-mit-Thrombozytopenie-Syndrom (TTS)
19.03.2021	Vaxzevria	Thrombose-mit-Thrombozytopenie-Syndrom (TTS)

### Veröffentlichung von Aufklärungsmerkblättern, Anamnese- und Einwilligungsbögen.

Vom Deutschen Grünen Kreuz e. V., Marburg, in Kooperation mit dem Robert Koch-Institut werden jeweils dem Stand der Wissenschaft angepasste Aufklärungsmerkblätter sowie Anamnese- und Einwilligungsbögen für COVID-19-Impfstoffe erstellt. Diese werden mit dem PEI abgestimmt. Die Dokumente werden laufend aktualisiert, um zu gewährleisten, dass Impfwillige stets aktuell über Nutzen und Risiko der jeweiligen COVID-19-Impfung informiert sind.

### Frequently Asked Questions (FAQ).

Vorgefertigte Botschaften in Form von häufig gestellten Fragen (FAQ) können bei der Beantwortung von Fragen hilfreich sein. FAQ auf den Webseiten des Paul-Ehrlich-Instituts [40] und des Robert Koch-Instituts [41] greifen dabei auch Fragen zur Sicherheit der COVID-19-Impfstoffe auf. Dabei werden Themen wie potenzielle Langzeitnebenwirkungen und Impfung bei bestehenden Allergien adressiert.

Eine offene Kommunikation auch möglicher Risiken ist eine Voraussetzung für eine hohe Impfakzeptanz in der Bevölkerung. Der Nutzen der COVID-19-Impfung für die Gesundheit Einzelner und der Bevölkerung sowie ihr Effekt im Kampf gegen die Pandemie hängen wesentlich vom Vertrauen in die Impfung ab. Um dem Rechnung zu tragen, informiert das Paul-Ehrlich-Institut über sicherheitsrelevante Themen in unterschiedlichen Formaten.

## Fazit und Ausblick

Die Beispiele zeigen, dass Risikokommunikation in erster Linie die gezielte, direkt an Fachkreise sowie an Patientinnen und Patienten gerichtete Information zu Anwendungsrisiken und deren Minimierung umfasst. Dabei ist je nach Anwendungsrisiko die Auswahl eines geeigneten Instruments von großer Bedeutung. Die Objektivierung der Effektivität einer Maßnahme bedarf der Überprüfung durch Studien bzw. durch geeignete Pharmakovigilanzforschungsprojekte.

Ergänzend wird der Austausch zwischen den Bundesoberbehörden und den Angehörigen der Heilberufe (vertreten durch deren Arzneimittelkommissionen und Fachgesellschaften) weiter intensiviert werden, um behördliche Kommunikation noch besser auf die praktischen Anforderungen des medizinischen Alltags anzupassen.

Die Bundesoberbehörden werden die Risikokommunikation auch aufgrund der Chancen, die die Digitalisierung bietet, zukünftig weiter stärken. Die verpflichtende, digitale Bereitstellung der Rote-Hand-Briefe und von Schulungsmaterial in Praxissoftwaresystemen ist durch entsprechende Änderungen des SGB V und des AMG gesetzlich geregelt und mit Frist zum 01.07.2023 umzusetzen, was als wichtiger Schritt im Sinne einer weiteren Verbesserung der Sicherheit von Patientinnen und Patienten anzusehen ist. Die Details der Umsetzung werden mit der nationalen Agentur für Digitale Medizin (gematik) im Rahmen der Telematikinfrastruktur diskutiert.

Die gezielte Kommunikation braucht ergänzend auch die Begleitung durch eine aktive Presse- und Öffentlichkeitsarbeit. Aktive Pressearbeit und die Pflege von Medienkontakten helfen, Risikoinformationen gezielt und schnell zu platzieren. Die Maßnahmen der Öffentlichkeitsarbeit zielen darauf ab, die Aufgaben und Ziele der Behörde in der Bevölkerung bekannt zu machen. Hier geht es auch darum, Vertrauen in deren Handeln zu schaffen. Die entsprechenden Kommunikationsplattformen müssen etabliert und akzeptiert sein, damit sie von der Bevölkerung genutzt werden und die Behörde als zuverlässige Quelle für Risikoinformationen wahrgenommen wird.

## Footnotes

1 www.bfarm.de/DE/Arzneimittel/Pharmakovigilanz/Risikoinformationen/Schulungsmaterial/_functions/Schulungsmaterial_Formular.html.

2 www.pei.de/DE/arzneimittelsicherheit/schulungsmaterial/schulungsmaterial-node.html.
